# Evaluating technical efficiency and influencing factors in traditional Chinese medicine hospitals: evidence from Hebei Province, China

**DOI:** 10.3389/fpubh.2025.1621755

**Published:** 2025-09-10

**Authors:** Ziyin Liao, Jun Zhou, Haochu Yang, Donglin Hou

**Affiliations:** Dong Fureng Economic and Social Development School, Wuhan University, Wuhan, China

**Keywords:** three-stage model, SFA model, Malmquist model, Tobit regression, hospital efficiency

## Abstract

**Background:**

The efficiency of public hospitals, particularly Traditional Chinese Medicine (TCM) hospitals, has emerged as a critical issue in China's healthcare reform, compounded by challenges such as low diagnostic revenue, regional disparities, and increasing adoption of Western medicine practices. As traditional Chinese medicine plays an indispensable role in managing chronic conditions among older adults, this study addresses critical healthcare challenges within China's aging population context. Therefore, this study aims to systematically analyze the technical efficiency of public TCM hospitals in Hebei Province.

**Methods:**

This research employs an integrated three-stage analytical framework using advanced efficiency measurement techniques to assess the technical efficiency of 21 public TCM hospitals in Hebei Province from 2014 to 2018. The methodology combines static efficiency evaluation with dynamic efficiency analysis, while regression modeling identifies key efficiency determinants. Specifically, data were collected from hospital records and analyzed using Data Envelopment Analysis, Stochastic Frontier Analysis, and Super Slack-Based Measure models.

**Results:**

Findings reveal that average technical efficiency of TCM hospitals in Hebei is suboptimal, primarily driven by insufficient pure technical efficiency rather than scale inefficiency. Environmental factors, including geographic location and local TCM practitioner training, significantly influence efficiency levels. Dynamic analysis indicates declining technological progress, counteracting marginal improvements in managerial efficiency. Furthermore, operational and financial factors show varied impacts: bed utilization rates, personnel expenditure ratios, and total asset turnover rates correlate positively, while depreciation costs, management expense ratios, and bed capacity demonstrate negative effects. Consequently, the research emphasizes that internal management optimization should take precedence over scale expansion.

**Conclusions:**

This research contributes to deeper understanding of efficiency dynamics in TCM hospitals through advanced analytical techniques. Most critically, policymakers should prioritize enhancing operational management and targeted resource distribution to achieve sustainable improvements in TCM hospital performance. Additionally, hospital management can benefit from adopting commitment-based practices to improve efficiency performance and optimize healthcare delivery outcomes.

## 1 Introduction

China's healthcare system faces mounting pressure to improve efficiency and quality amid rising demand for medical services, with public hospitals constituting over 60% of all healthcare institutions according to the 2020 National Health Statistical Yearbook. Within this context, Traditional Chinese Medicine (TCM) hospitals represent a unique challenge, as they must balance preservation of traditional medical heritage with modern healthcare efficiency requirements. Ownership structure also shapes efficiency patterns in China's hospital sector. A DEA-based comparison of public and private hospitals in Beijing found that private hospitals generally exhibited lower technical efficiency but higher scale efficiency than public hospitals, suggesting different managerial and scale drivers across ownership types (1). Recent national analyses show that a higher share of private hospitals is associated with improved structural efficiency by expanding capacity and service output, while hospital-level comparisons around COVID-19 highlight ownership-specific efficiency and profitability patterns (2, 3). This provides a useful benchmark for situating the performance of Hebei's public TCM hospitals within the broader system.

TCM hospitals face a critical efficiency challenge in China's healthcare system, as they must balance their dual-service model—integrating traditional practices such as acupuncture, herbal medicine, and tuina with advanced Western medical technologies—while addressing mounting operational pressures including slow treatment effects, complex preparation methods, declining traditional practices, and growing patient preference for Western medicine, all of which directly impact their technical efficiency and sustainability. These institutions offer comprehensive healthcare services, including diagnosis and treatment of chronic diseases, post-surgical rehabilitation, preventive care, and health maintenance, emphasizing holistic approaches to patient care that address physical, mental, and environmental factors. The COVID-19 pandemic further underscored this efficiency imperative by demonstrating TCM's clinical value in pain management, inflammation reduction, and fracture healing, yet simultaneously exposing the critical importance of optimizing TCM hospital operations to maintain service delivery effectiveness within modern healthcare systems under crisis conditions, creating an urgent need for systematic efficiency evaluation and improvement strategies.

Recognizing these challenges, the central government has introduced policies to support TCM. The “Traditional Chinese Medicine Development Strategic Plan (2016–2030)” aims to enhance TCM hospital operations, upgrade grassroots infrastructure, and expand global influence. The “Traditional Chinese Medicine Law” (2017) and the “Several Policy Measures for Accelerating the Development of TCM” (2021) reinforced legal and policy frameworks, ensuring sustainable growth. The “14th Five-Year Plan for TCM Development” (2022) positioned TCM as a key element of China's socialist development strategy. Despite these efforts, regional disparities, inefficient management, and resource allocation issues continue to hinder progress, particularly in Hebei Province.

This study addresses a critical research gap by systematically evaluating the technical efficiency of public TCM hospitals in Hebei Province, where institutions struggle with regional disparities, outdated management practices, and lower TCM treatment revenues compared to Western medicine departments. While previous studies have examined hospital efficiency broadly, few have focused specifically on TCM hospitals' unique operational characteristics and efficiency determinants within China's evolving healthcare landscape. To fill this gap, this research evaluates the technical efficiency of public TCM hospitals in Hebei using advanced methods, including Data Envelopment Analysis (DEA), Stochastic Frontier Analysis (SFA), and Super Slack-Based Measure (SBM). The study also employs the Malmquist Index and Tobit models to analyze efficiency dynamics and influencing factors. By providing comprehensive insights into TCM hospital operational challenges and efficiency patterns, this study contributes evidence-based recommendations for policymakers and hospital administrators seeking to enhance TCM hospital performance within national healthcare reforms.

The paper is structured as follows: Section 2 reviews the existing literature, while Section 3 outlines the theoretical framework. Section 4 presents the methodology, including data resource, static efficiency analysis results and dynamic analysis. Finally, Section 5 summarizes the findings and offers policy recommendations for improving TCM hospital efficiency in Hebei Province.

## 2 Literature review

### 2.1 International healthcare resource allocation models

Research on healthcare resource allocation has primarily focused on countries like the UK, US, Germany, and Singapore. The UK's National Health Service (NHS) pioneered nationwide resource planning, ensuring equitable distribution based on demand (4). The US follows a market-driven system shaped by political, cultural, and economic factors (5). Germany employs a public, non-profit-based model with strict regulations on hospital investments and private practitioners (6). Singapore, constrained by limited resources and population, has developed a four-tier system resembling aspects of China's approach (7).

### 2.2 TCM hospital resource allocation in China

In China, TCM human resource allocation has gained attention, with Xi Jinping emphasizing its role in healthcare innovation and service delivery. However, studies (8, 9) reveal imbalances, including a surplus of Western medicine practitioners and a shortage of TCM professionals. The expansion of tertiary hospital beds has led to diminishing scale returns, squeezing primary healthcare, and hindering tiered treatment policies. Recent research by Xu et al. (10) and Lin and Lai (11) further confirms these resource allocation challenges, highlighting the need for systematic efficiency evaluation in TCM hospitals. Research (12, 13) highlights rapid asset and liability growth in TCM hospitals, increasing debt risks. The accelerated expansion of tertiary hospital beds further strains resource efficiency.

### 2.3 DEA applications in hospital efficiency studies

Efficiency measurement techniques, particularly Data Envelopment Analysis (DEA) and Stochastic Frontier Analysis (SFA), are widely applied globally. Early foundational work by Charnes et al. (14) established DEA as a non-parametric method for efficiency measurement, while Banker et al. (15) introduced the BCC model to account for variable returns to scale. Studies by Staat (16) and Barros et al. (17) used DEA to assess hospital efficiency, considering bed numbers and staff as inputs and inpatient numbers as outputs. More recent applications by Kohl et al. (18) and Xiao et al. (19) have demonstrated the evolution of DEA methodologies in healthcare settings, incorporating advanced models such as three stage DEA to capture complex hospital operations.

Other research (20, 21) explored Super-Efficiency Models and panel data, revealing lower efficiency in healthcare sectors affected by income disparities. Tone (22) introduced the Slack-Based Measure (SBM) model, which has become increasingly popular for hospital efficiency evaluation due to its ability to handle undesirable outputs and provide more accurate efficiency scores.

### 2.4 DEA applications in TCM hospital context

Küçük et al. (23) analyzed healthcare reforms' impact on hospital efficiency in Turkey using DEA, finding higher efficiency in larger hospitals. Similarly, Fumbwe et al. (24) applied DEA in Tanzania, advocating resource redistribution for better efficiency. In China, studies (25, 26) have used DEA and SFA to enhance public hospital efficiency. Recent comprehensive study by Yang et al. (27) has applied advanced DEA variants including network DEA to evaluate TCM hospital efficiency, revealing significant regional disparities and management inefficiencies.

### 2.5 Dynamic efficiency studies and Malmquist index applications

Dynamic efficiency analysis has gained prominence in hospital efficiency research. Caves et al. (28) introduced the Malmquist productivity index, which has been extensively applied to track efficiency changes over time. Färe et al. (29) further developed the methodology by decomposing the Malmquist index into technical efficiency change and technological progress components. Recent applications in healthcare such as Wang et al. (30) have demonstrated the value of dynamic analysis in understanding hospital performance trends and identifying factors driving efficiency changes.

However, research on TCM hospital efficiency remains limited. Studies (31) indicate challenges such as low efficiency due to inadequate human resources and suboptimal asset use. Sun et al. (32) has begun to address this gap by applying super-SMB specifically to TCM hospitals, but their studies are limited in scope and do not employ comprehensive multi-stage analytical frameworks.

### 2.6 Research gaps and study positioning

Despite the extensive application of DEA in hospital efficiency studies, several critical gaps remain in the literature. First, most studies focus on general hospitals or Western medicine institutions, with limited attention to TCM hospitals' unique operational characteristics and dual-service models. Second, while static efficiency analysis is common, few studies integrate static and dynamic approaches with environmental factor adjustments. Third, regional-level analysis of TCM hospital efficiency, particularly in economically diverse provinces like Hebei, remains underexplored.

DEA remains the primary tool for evaluating hospital efficiency, assessing resource utilization, and operational effectiveness. Advanced models like DEA-Tobit and DEA-Malmquist enable dynamic performance tracking. While China's research has focused on national public and private hospitals, regional studies, especially on TCM hospitals, are scarce. This study addresses these gaps by employing an integrated three-stage analytical framework that combines static efficiency evaluation, dynamic performance tracking, and comprehensive environmental factor analysis specifically tailored to TCM hospital operations in Hebei Province.

A comprehensive review of domestic and international literature (33, 34) identified key input and output variables in hospital efficiency studies. The five most common inputs are hospital beds, fixed assets, non-medical staff, medical staff, and operational costs, while the top outputs include inpatient numbers, surgical procedures, emergency visits, outpatient visits, and average hospital stay. These variables are widely used in DEA applications to evaluate hospital efficiency (35).

To enhance methodological rigor and address the identified research gaps, this study extends the traditional DEA framework by integrating multiple methodologies. SFA is used to control for environmental influences, while the super-SBM method overcomes DEA's inherent limitations. The Malmquist index method decomposes efficiency changes from 2014 to 2018, and Tobit regression identifies key factors affecting efficiency. This multi-method approach ensures a comprehensive and robust evaluation of hospital efficiency.

## 3 Methodology

### 3.1 Principle of the modified three-stage DEA model

The selection of our integrated DEA-SFA-Super SBM analytical framework is strategically justified by the complementary strengths of each methodology and their proven effectiveness in healthcare efficiency analysis. This methodological preference addresses specific limitations inherent in single-method approaches while leveraging the synergistic benefits of combined techniques (33, 34).

**Figure 1:**
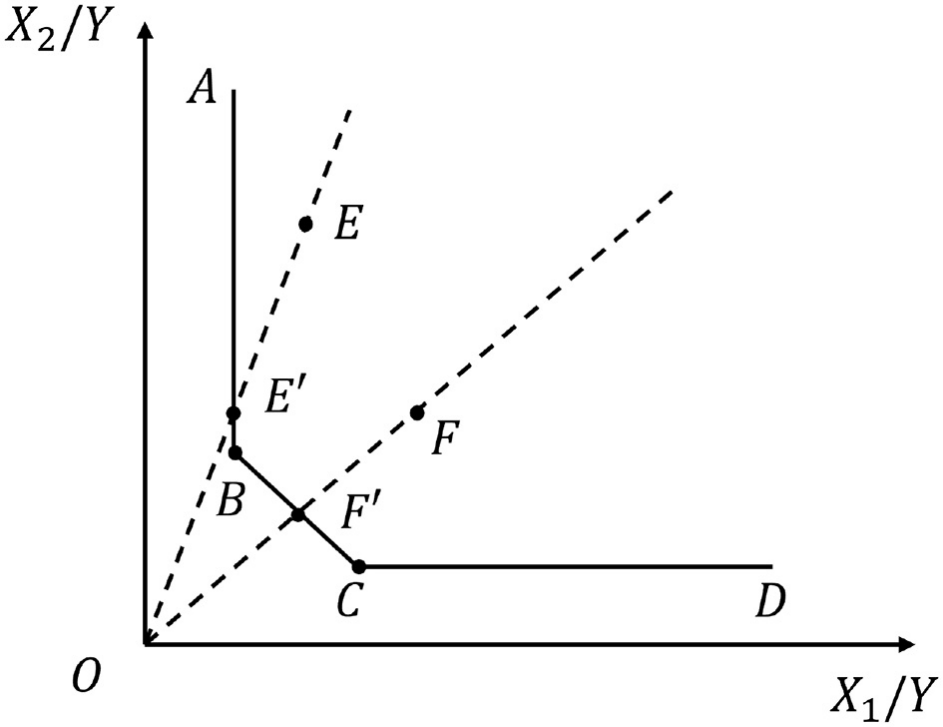
The DEA model determines the optimal input-output ratio based on the efficient frontier.

The traditional DEA method, introduced by Charnes et al. (14), is a non-parametric frontier analysis using linear programming to evaluate multiple DMUs. DEA was selected as our primary efficiency measurement tool because it is particularly suitable for multi-input, multi-output organizations like hospitals without requiring predetermined functional forms, making it ideal for capturing the complex production relationships in healthcare settings (36). It measures input-output ratios to construct a production frontier and determines each unit's efficiency based on its deviation from this frontier. Figure 1 illustrates the traditional DEA model, where *X* and *Y* represent input and output indicators. Points *B* and *C* lie on the efficient frontier, while points *E* and *F* represent inefficient units. According to Figure 1, units on the frontier have an efficiency score of 1. For inefficient unit *E*, the *OE* ray intersects the efficient frontier at *E*′, making its efficiency the ratio of *OE*′ to *OE*. The same applies to point *F*. To improve *E* to the efficient point *B* while maintaining output, inputs must be reduced. First, *E* should adjust to *E*′, and then, by further reducing input *X*_2_, it can reach the target efficiency point.

The three-stage DEA model enhances efficiency analysis through three phases: first, classic DEA calculates technical efficiency using input-output indicators; second, Stochastic Frontier Analysis (SFA) evaluates environmental and random factors; third, SFA-adjusted inputs are reintroduced into DEA for reevaluation. Comparing initial and final efficiency values reveals the impact of these factors. SFA integration is crucial as it addresses a fundamental limitation of traditional DEA—its inability to separate environmental influences from managerial inefficiency, which can lead to biased efficiency assessments (37, 38). SFA is crucial as it removes biases from environmental and random influences, isolating the effects of management and input variables.

Originally proposed by Fried et al. (37), this method has been applied in taxation, banking, and other fields to refine efficiency assessments by eliminating random factors. In China, three-stage DEA is still emerging in hospital efficiency research, with most studies relying on traditional DEA. However, a limitation arises when multiple units on the frontier exhibit efficiency variations. To address this ranking problem and provide more discriminatory power, we integrate the Super-SBM model in the third stage, which overcomes the limitation of traditional DEA when multiple DMUs achieve perfect efficiency scores and enables more realistic efficiency measurements by accounting for input excesses and output shortfalls independently (22, 35). To address this, we refine the traditional model by integrating the super-efficiency SBM model in the third stage, improving measurement accuracy and strengthening the foundation for efficiency analysis.

### 3.2 Design of the modified three-stage model

#### 3.2.1 CCR model

The CCR model is designed to measure technical efficiency (TE) and can extend single-output efficiency to multi-output efficiency. While similar to the BCC model in DEA, the CCR model differs in its approach. Technical efficiency in the CCR model is the overall efficiency, which can be decomposed into scale efficiency (SE) and pure technical efficiency (PTE). PTE indicates the level of resource utilization, while SE reflects changes in scale returns due to increased inputs (14). Let there be n DMUs, for m inputs and s outputs. For the k-th DMU:

Input vector:


(1) $$x_{k}={\left(x_{1k},x_{2k},\ldots ,x_{mk}\right)}^{T}.$$

Output vector:


(2) $$y_{k}={\left(y_{1k},y_{2k},\ldots ,y_{sk}\right)}^{T}.$$

The CCR model solves for output weights u, and input weights v to maximize the efficiency θ_*k*_:


(3) $$\begin{array}{l} \text{u}={\left(u_{1},u_{2},\ldots ,u_{s}\right)}^{T}, \end{array}$$

(4) $$\begin{array}{l} \text{v}={\left(V_{1},V_{2},\ldots ,V_{M}\right)}^{T}. \end{array}$$

Function:


(5) $$\begin{array}{l} \max\theta_{k}=\frac{\sum_{r=1}^{s}u_{r}y_{rk}}{\sum_{i=1}^{m}v_{i}x_{ik}}. \end{array}$$

Constraints:


(6) $$\begin{array}{r} \frac{\sum_{r=1}^{s}u_{r}y_{rj}}{\sum_{i=1}^{m}v_{i}x_{ij}}\leq 1,\;\forall j=1,2,\ldots ,n\\ u_{r}\geq 0,\;\forall r=1,2,\ldots ,s\\ v_{i}\geq 0,\;\forall i=1,2,\ldots ,m \end{array}$$

#### 3.2.2 BCC model

The BCC model is a DEA model first developed by Banker (39) as a measure of pure technical efficiency by introducing the concept of the distance function (40). The BCC model overcomes the assumption of constant returns to scale in the CCR model by assuming variable returns to scale and reflects both pure technical efficiency and efficiency of scale (15).

The BCC model, which assumes variable returns to scale and is a model that only considers pure technical efficiency (PTE) that excludes scale-efficiency, is a CCR model with the addition of the constraint $\sum_{j=1}^{n}{\text{λ}}_{j}=1$. The optimization constraint equation is as follows:


(7) $$\begin{array}{l} \mathrm{Min}\theta_{j_{0}}-\varepsilon \left({ê}^{t}S^{-}+e^{t}S^{+}\right), \end{array}$$

(8) $$\text{s.t. }\{\begin{array}{c} \sum_{j=1}^{n}{\text{λ}}_{j}y_{r_{j}}-s^{+}=y_{rj_{0}}\\ \sum_{j=1}^{n}{\text{λ}}_{j}x_{ij}+s^{-}=\theta_{j_{0}}x_{ij_{0}}\\ \sum_{j=1}^{n}{\text{λ}}_{j}=1,{\text{λ}}_{j}\geq 0 \end{array},$$

where *s*^+^ and *s*^−^ represent input and output slack variables, respectively. The CCR model calculates the technical efficiency (TE) of a DMU, while the BCC model isolates the impact of scale, yielding pure technical efficiency (PTE). Scale efficiency is then derived by dividing TE by PTE, with a standardized threshold of 1 under the BCC model.

This study evaluates economies of scale by analyzing increasing, decreasing, and constant returns to scale. When all inputs grow proportionally, output expands at the same rate under constant returns to scale. If output grows at a slower rate, returns to scale decrease; if it grows faster, returns to scale increase. Increasing returns to scale indicate economies of scale, while decreasing returns signal dis-economies of scale. In hospital efficiency analysis, institutions with decreasing returns should downsize, whereas those with increasing returns should enhance efficiency by expanding key assets (41).

#### 3.2.3 SFA model

Fried et al. (37) argues that DMU efficiency is influenced by operational inefficiencies, environmental factors, and statistical noise, necessitating SFA regression analysis to separate these effects. Originally introduced by Aigner et al. (42), SFA has been widely applied in empirical research. Kumbhakar and Lovell (43) further developed the methodology by demonstrating how SFA can effectively separate managerial inefficiency from environmental influences in healthcare settings.

In practical terms, the SFA model serves as a “filter” that helps us understand why some hospitals appear inefficient. When we observe that a hospital is not performing optimally, this could be due to three reasons: poor management decisions (true inefficiency), unfavorable external conditions such as geographic location or patient demographics (environmental factors), or simply random events like equipment breakdowns or staff shortages (statistical noise). The SFA model mathematically separates these three components, allowing us to identify which hospitals are genuinely underperforming due to management issues vs. those facing external challenges beyond their control.

In the SFA model, the error term comprises two components: random error (*v*) and technical inefficiency (*u*). Typically, *v* follows a normal distribution, while *u* is a non-negative random variable. This decomposition enables the precise estimation of DMU technical efficiency.

In the first stage, the slack variable (*s*_*ni*_) represents the deviation from optimal efficiency. SFA regression employs input factors as dependent variables and environmental factors as independent variables, modeled as:


(9) $$\begin{array}{l} s_{ni}=f\left(z_{i},\beta_{n}\right)+v_{ni}+u_{ni}, \end{array}$$

where *s*_*ni*_ is the slack variable, *f*(*z*_*i*_, β_*n*_) denotes environmental impact, *v*_*ni*_ represents random error, and *u*_*ni*_ indicates technical inefficiency. With *k* environmental variables, *z*_*i*_ = *z*_1*i*_ + *z*_2*i*_ + ⋯ + *z*_*ki*_, and β_*n*_ as their corresponding parameters. Here, $v_{ni}\sim N\left(0,\sigma_{v}^{2}\right)$, and *u*_*ni*_ follows a truncated normal distribution. These two terms are assumed to be independent.

If the variance of management inefficiency is $\sigma_{v}^{2}$, and the variance of the random disturbance term is $\sigma_{u_{n}}^{2}$, satisfying $\gamma =\frac{\sigma_{v}^{2}}{\sigma_{v}^{2}+\sigma_{u_{n}}^{2}}$, then as γ approaches 1, the management factor *S*_*in*_ is the main influencing factor. As γ approaches 0, the random factor is the main influencing factor. The formula for separating management inefficiency is as follows:


(10) $$\begin{array}{l} E\left(\mu |\varepsilon \right)=\sigma_{\sigma }\left[\begin{array}{c} \frac{\phi \left(\text{λ}\frac{\varepsilon }{\sigma }\right)}{\text{Φ}\left(\text{λ}\frac{\varepsilon }{\sigma }\right)}+\text{λ}\frac{\varepsilon }{\sigma } \end{array}\right], \end{array}$$

where $\sigma_{\sigma }=\frac{\sigma_{u}\sigma_{v}}{\sigma }$, $\sigma =\sqrt{\sigma_{u}^{2}+\sigma_{v}^{2}}$, and $\text{λ}=\frac{\sigma_{u}}{\sigma_{v}}$. Thus, the random error term serves as a mechanism for relaxing variables, reducing environmental impact to decrease the value of management inefficiency.

The practical significance of this adjustment process can be understood through a simple analogy: imagine comparing the performance of two hospitals, one located in an affluent urban area with abundant medical resources and another in a remote rural region with limited infrastructure. Without SFA adjustment, the rural hospital might appear less efficient simply due to its challenging operating environment rather than poor management. The SFA correction essentially “levels the playing field” by adjusting for these environmental differences, allowing us to make fair comparisons of managerial efficiency across different operating contexts.

Coefficient significance in the SFA model is crucial for adjusting slack variables. A negative coefficient reduces slack and enhances efficiency, while a positive coefficient increases slack, lowering efficiency. Even if insignificant, coefficient values influence input variable trends. Based on SFA results, DMU input values are corrected to account for environmental and noise effects, ensuring a more accurate efficiency assessment.

The adjusted input value (*x*_*ni*_) is computed as:


(11) $$\begin{array}{l} x_{ni}=x_{ni}+\left[\underset{i}{\max}\left(z_{ip}\right)-z_{ip}\right]+\left[m\left(x_{i}\left(v_{ni}\right)-v_{ni}\right)\right], \end{array}$$

where $\underset{i}{\max}\left(z_{ip}\right)-z_{ip}$ standardizes external environmental conditions, and *m*(*x*_*i*_(*v*_*ni*_) − *v*_*ni*_) ensures uniform random error adjustments. This adjustment process transforms the original input data into “environmentally-neutral” values, enabling more accurate efficiency comparisons by removing the influence of factors beyond management control. The resulting adjusted inputs provide a cleaner foundation for subsequent efficiency analysis using the Super-SBM model.

#### 3.2.4 Super-SBM model

While traditional DEA models provide valuable insights into hospital efficiency, they face significant limitations when multiple hospitals achieve perfect efficiency scores of 1.0, making it impossible to rank or differentiate among the best performers. Additionally, standard DEA models assume proportional changes in all inputs and outputs, which may not reflect the complex reality of hospital operations where different resources can be adjusted independently. To address these limitations and build upon the environmentally-adjusted inputs from the SFA stage, this study employs the Super-SBM model, which offers enhanced discriminatory power and more realistic efficiency measurements.

**Figure 2:**
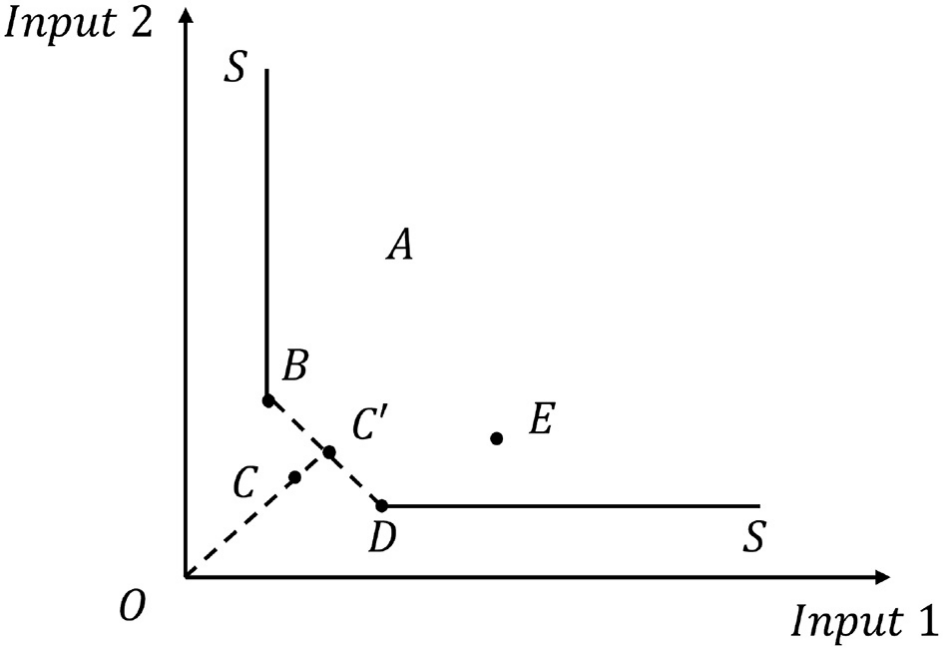
Explanation of the Super-SBM model principle and application in distinguishing DMUs on the efficient frontier.

In the CCR and BCC models, all outputs are proportionally expanded, or inputs are contracted by a common factor along the input direction. Anderson's super-efficiency model addresses the issue of all DMUs being on the production frontier (44). Tone and Tsutsui (45) extended this approach by developing the Super-SBM model specifically to handle slack variables and provide more nuanced efficiency measurements. The principle is explained in Figure 2, where points B, C, and D are on the optimal frontier, and it is impossible to measure the efficiencies of these three DMUs. By constructing a super-efficiency model, the efficiencies of the DMUs on the optimal frontier can be excluded from the model while the efficiencies of the DMUs on the optimal frontier can be measured, for example, by excluding point C from the optimal point, we can see that a new frontier BC'D is formed, and the efficiency value of point C, which has been excluded, is the new frontier BC'D. The efficiency value of point C, which has been excluded, is the new frontier BC'D, which has been excluded. The efficiency value of the excluded point C is the ratio of the distance from OC' to the distance from OC on the new frontier, which is greater than one, so point C was originally on the original efficient frontier, and can still be made more efficient by increasing the input values of *X*_1_ and *X*_2_ compared to the present. The traditional DEA approach treats all inputs and outputs as proportionally adjustable, but this assumption fails to capture the reality that hospitals can independently adjust different resources (such as staff, beds, or equipment) without maintaining fixed ratios. The Super-SBM model overcomes this limitation by explicitly accounting for input excesses and output shortfalls, providing a more realistic assessment of hospital efficiency.

Based on the super-efficiency model (22), the Super-Slack Measurement (Super-SBM) compensates for the shortcomings of the BCC model. The Super-SBM model deals with input excesses and output deficiencies and gives a scalar measure of all inefficient units by using an additive model (46). Sun et al. (32) demonstrated the superior performance of Super-SBM in healthcare applications, particularly for ranking efficient hospitals and identifying specific areas for improvement. The Super-SBM model can be expressed as:


(12) $$\begin{array}{l} \delta^{*}=\min\frac{\frac{1}{m}\sum_{i=1}^{m}\bar{x}/x_{i0}}{\frac{1}{s}\sum_{r=1}^{s}ȳ/y_{r0}}, \end{array}$$

(13) $$\text{ s.t. }\{\begin{array}{l} \bar{x}\leq \sum_{j=1,\neq 0}^{n}{\text{λ}}_{j}x_{j}\\ \bar{y}\leq \sum_{j=1,\neq 0}^{n}{\text{λ}}_{j}y_{j}\\ \bar{x}\geq x_{0},\bar{y}\leq y_{0}\\ \text{λ}\geq 0 \end{array}.$$

The integration of SFA-adjusted inputs with the Super-SBM model creates a powerful analytical framework that first removes environmental bias through SFA corrections and then applies sophisticated efficiency measurement through Super-SBM. This two-stage approach ensures that efficiency scores reflect genuine managerial performance rather than external circumstances, while providing the discriminatory power needed to rank and compare hospital performance effectively.

### 3.3 Malmquist exponential modeling

When CCR and BCC models are added to consider the time change, the panel data will cause the optimal frontier of each period to be different, which makes the comparison of the efficiency value of each period lack uniform standards, therefore, the traditional DEA is only suitable for static research under the cross-section data (47). The Malmquist exponential model can make up for the above drawbacks very well. This model was proposed by Malmquist (48), and now we widely use the model proposed by Färe et al. (29). Based on the DEA model, he further decomposed the total factor productivity and established the Malmquist index to decompose and analyze the changes in efficiency dynamically. It is expressed in the following formula, and the letters are defined in Table 1:


(14) $$\begin{array}{l} TC={\left[\frac{D^{t}\left(x_{t+1},y_{t+1}\right)}{D^{t+1}\left(x_{t+1},y_{t+1}\right)}\times \frac{D^{t}\left(x_{t},y_{t}\right)}{D^{t+1}\left(x_{t},y_{t}\right)}\right]}^{\frac{1}{2}}, \end{array}$$

(15) $$\begin{array}{l} EC=\frac{D^{t+1}\left(x_{t+1},y_{t+1}\right)}{D^{t}\left(x_{t},y_{t}\right)}, \end{array}$$

(16) $$\begin{array}{l} TFP=\text{EC}\times \text{TC}=\text{PE}\times \text{SE}\times \text{TC}. \end{array}$$

**Table 1:** Total factor productivity formula definitions.

**Abbreviation**	**Definition**
TFP	Total factor productivity change index
EC	Index of change in technical efficiency
TC	Index of change in technical progress
PE	Index of change in pure technical efficiency
SE	Change in scale efficiency Index

## 4 Data and analysis

### 4.1 Data sources

This study focuses on 21 representative public Chinese medicine hospitals in 11 prefectural-level cities in Hebei Province. The selection criteria are: first, representativeness, ensuring the hospitals reflect the local development of Chinese medicine; second, data availability, with accurate and complete data from relevant hospital databases, free from missing or erroneous values; and third, extensive sample coverage, considering provincial, municipal, and county hospitals to examine how hospital efficiency varies across levels and locations. Additionally, macroeconomic data are sourced from the Hebei Provincial Health and Family Planning Commission's Financial Statistics Annual Report, the Hebei Provincial Health Statistics Yearbook, the China Health Statistics Yearbook, and the Hebei Provincial Statistical Information Website, along with data from the Planning and Finance Department, the Information Statistics Center, and the Hebei Provincial Public Hospital Information Center.

### 4.2 Variable setting

The selection of variables is the first step in analyzing hospital technical efficiency. Changes in input and output variables affect efficiency calculations. Hospitals, like other industries, invest capital and labor to provide medical services, but quantifying service output is challenging, requiring multiple variables. Thus, it is more suitable to design models with multiple output correspondences (49). The most recommended approach is to use variables from existing studies on similar topics. In studying technical efficiency, the goal is to examine how hospitals coordinate resource elements to improve resource utilization and maximize service output at the current technological level (50).

The inclusion of both resource and financial indicators in this study is explicitly justified by the dual nature of hospital performance evaluation. Resource efficiency measures capture the physical transformation process of converting inputs (staff, beds, equipment) into healthcare services (patient treatments), reflecting operational effectiveness and clinical productivity. Financial efficiency measures, conversely, assess the economic sustainability and revenue generation capacity of hospitals, which is particularly critical for TCM hospitals operating within China's healthcare payment systems (33). This dual-perspective approach is essential because hospitals must simultaneously optimize clinical outcomes and financial viability to ensure long-term sustainability (34). For TCM hospitals specifically, this distinction is crucial as they face unique challenges in balancing traditional treatment methods with modern healthcare economics, where TCM services often generate lower revenues compared to Western medicine procedures (51).

Based on the literature review of medical institutions, input and output variables were selected from the perspectives of resource and financial efficiencies, as shown in Appendix Table A1. For resource efficiencies, input variables include the number of healthcare personnel, beds, and fixed assets, representing human, material, and financial resources. Output variables include the number of outpatients and inpatients, reflecting hospital workload. Additionally, financial efficiency was analyzed with output variables like hospitalization and consultation revenues, along with other major indicators.

For the selection of environmental variables in the second stage of the SFA methodology, we also need to take into account the external disturbances to the operation of TCM hospitals. The traditional DEA model has no way to exclude the influence of external factors and random factors on the efficiency measurement; for example, provincial hospitals in provincial capitals will inevitably receive more financial care, and excellent talents tend to gather in the central city. The talent resources in big cities will be more high-quality. If we do not exclude these factors, we will not be able to effectively measure the influence of the hospitals' decision-making and management on the technical efficiency of the hospitals (1). After fully understanding and considering the actual situation of Chinese medicine hospitals in Hebei Province, we selected Science Education inputs and External financial inputs as representative environmental variables.

Table 2 presents a comprehensive overview of the complete analytical framework employed in this study, illustrating the integration of input variables, output variables, environmental factors, and the three-stage analytical process. This framework demonstrates how resource and financial indicators are systematically incorporated across different stages of the analysis to provide a holistic assessment of TCM hospital efficiency.

**Table 2:** Complete analytical framework for TCM hospital efficiency analysis.

**Analysis stage**	**Variable category**	**Specific variables**
Stage 1: DEA analysis	Resource inputs	Number of healthcare personnel,
		Number of beds, Fixed assets
	Resource outputs	Number of outpatients, Number of inpatients
	Financial outputs	Hospitalization revenue, consultation revenue,
		Total operating revenue
Stage 2: SFA adjustment	Environmental variables	Science education inputs,
		External financial inputs
	Adjustment process	Slack variable regression,
		Environmental factor separation
Stage 3: Super-SBM analysis	Adjusted inputs	Environmentally-adjusted resource inputs
	Efficiency measures	Technical efficiency, pure technical efficiency and scale efficiency
	Dynamic analysis	Malmquist productivity index,
		Technical change, efficiency change
	Determinant analysis	Tobit regression with operational
		and financial factors

### 4.3 Descriptive statistics

By the end of the 13th Five-Year Plan, over 1,900 national medical halls had been established in Hebei, with nearly 90% of primary healthcare sites offering TCM services. Resident satisfaction with TCM care has continuously increased. Hebei currently has 148 national and provincial key TCM specialties and 34 inheritance studios of renowned TCM practitioners, working to pass on TCM skills and culture. Policy-wise, the Province has standardized TCM clinical departments in hospitals, improved service quality, promoted TCM-Western medicine integration, and strengthened TCM's role in epidemic prevention and treatment while expanding TCM talent and fostering leading practitioners.

As of the end of 2018, Hebei had 1,467 general hospitals and 243 TCM hospitals, with general hospitals being six times more numerous. From 2015 to 2018, the number of general hospitals grew by 28.9%, while TCM hospitals grew by 7%. Bed utilization rates in 2018 were 83.8% for general hospitals and 79.59% for TCM hospitals, a decline for general hospitals from 2015, while TCM hospitals showed improvement, utilizing beds more efficiently.

Regarding human resources, as shown in Appendix Tables A2, A3, TCM hospitals had fewer employees than general hospitals, with managerial staff being particularly limited. Healthcare technicians made up 84.67% of the workforce in TCM hospitals, a higher proportion compared to larger general hospitals.

Table 3 shows an analysis of bed resources in the two hospitals. The average number of beds in general hospitals is 1.61 times higher than in TCM hospitals. Overall, both hospitals have relatively low numbers of bed managers, with general hospitals having 1.23 compared to 1.30 in TCM hospitals, indicating that TCM hospitals invest more human resources in bed management. The total fixed assets per bed are 53.85 in general hospitals, slightly higher than 52.59 in TCM hospitals, showing that while general hospitals have more beds, the fixed assets per bed are higher in TCM hospitals (52).

**Table 3:** Allocation of bed resources in general hospitals and TCM hospitals in Hebei Province.

**Indicators**		**General hospitals**	**TCM hospitals**
Average number of open beds	Average	2,679.39	1,665.24
	Standard deviation	1,331.14	646.32
	Maximum value	6,347.00	2,814.00
	Minimum value	850	774
Number of in-service staff for bed management	Average	1.23	1.3
	Standard deviation	0.23	0.25
	Maximum value	1.4	1.38
	Minimum value	1.13	1.2
Total fixed assets of beds (10,000 yuan)	Average	53.85	52.59
	Standard deviation	16.04	15.5
	Maximum value	90.9	90.1
	Minimum value	31.2	30.6

The number of open beds is 1.61 times higher in general hospitals than in TCM hospitals (2,679.39 vs. 1,665.24).

The number of in-service staff for bed management is slightly lower (1.23 vs. 1.30).

The total fixed assets of beds are slightly higher in general hospitals (53.85 vs. 52.59).

Table 4 shows that in terms of revenue, general hospitals are 2.4 times higher than TCM hospitals. The total expenditure of general hospitals is much higher than that of Chinese medicine hospitals, 94.43 for the former and 38.19 for the latter, which is 2.5 times that of the latter, and the overall income expenditure multiplier matches that of the latter. However, the surplus of public hospitals is 6.83, which is only 1.88 times higher than that of the latter (3.63), not in line with the multiplier ratio of income to expenditure. It can be seen that in terms of financial and economic profitability, Chinese medicine hospitals perform better than general hospitals. The average annual income of hospital staff is 68.75 in the former and 33.67 in the latter, which is 2.04 times higher than the average annual income of the latter. There is also a certain gap between the overall incentives for medical and nursing staff in TCM hospitals and their surpluses, as lower salaries and compensation will affect the motivation of medical and nursing staff and reduce the efficiency of their management (53).

**Table 4:** Income and expenditure of general hospitals and TCM hospitals in Hebei Province.

**Indicators**		**General hospitals**	**TCM hospitals**
Total income (10,000,000 yuan)	Average	101.25	41.82
	Standard deviation	71.8	12.91
	Maximum value	281.2	65.14
	Minimum value	28.5	21.53
Total expenditure (10,000,000 yuan)	Average	94.43	38.19
	Standard deviation	67.69	12.59
	Maximum value	264.33	59.93
	Minimum value	26.79	11.27
Net income (10,000,000 yuan)	Average	6.83	3.63
	Standard deviation	8.52	5.14
	Maximum value	40.73	21.03
	Minimum value	–10.63	–10.63
Average annual income of staff (10,000 yuan)	Average	68.75	33.67
	Standard deviation	53.36	48.84
	Maximum value	194.82	241.82
	Minimum value	65.02	15.33

Total income is 2.42 times higher in general hospitals than in TCM hospitals (101.25 vs. 41.82). Total expenditure is 2.47 times higher (94.43 vs. 38.19).

Net income is 1.88 times higher (6.83 vs. 3.63).

The average annual income of staff is 2.04 times higher (68.75 vs. 33.67).

Table 5 analyzes the provision of medical services in both countries. The number of consultations is 1.64 times higher, 87.60, than the number of consultations, 53.44. However, the number of doctors per capita per day is 5.78 for the former, which is seriously lower than the latter's 8.80, and the number of hospital discharges is 5.42 for the former, which is higher than that of the latter's 2.03, or 2.67 times, but the number of doctors per capita per day is 0.28 for the former, which is higher than that of the latter's 0.19, or 1.47 times, which reflects the relative lack of medical and nursing resources in TCM hospitals.

**Table 5:** Provision of medical services in general hospitals and traditional Chinese medicine hospitals in Hebei Province.

**Indicators**		**General hospitals**	**TCM hospitals**
Number of consultations (10,000)	Average	87.6	53.44
	Standard deviation	85.27	29.59
	Maximum value	378.55	109.25
	Minimum value	11.23	15.82
Number of hospital discharges (10,000)	Average	5.42	2.03
	Standard deviation	3.68	1.08
	Maximum value	21.58	5.14
	Minimum value	1.34	0.55
Number of consultations per doctor	Average	5.78	8.8
	Standard deviation	2.87	2.18
	Maximum value	15.63	12.92
	Minimum value	2.76	4.63
Number of discharges per doctor	Standard deviation	0.18	0.1
	Maximum value	0.74	0.35
	Minimum value	0.04	0.05

The number of consultations is 1.64 times higher in general hospitals than in TCM hospitals (87.60 vs. 53.44).

The number of hospital discharges is 2.67 times higher (5.42 vs. 2.03).

The number of consultations per doctor is 1.52 times lower (5.78 vs. 8.80).

The number of discharges per doctor is 1.47 times higher (0.28 vs. 0.19).

In conclusion, public TCM hospitals in Hebei Province have distinct characteristics. While they may have fewer departments, smaller scales, and fewer assets than general hospitals, national guidelines require that the rate of TCM treatment in outpatient clinics should not fall below 85%, and inwards, it should not be less than 70%. TCM hospitals should focus on specialization, integrating TCM and modern medicine to treat diseases and address chronic conditions effectively. However, TCM treatment does not require hospitalization, as it offers higher efficiency and output with less investment. With China strengthening its commitment to TCM, the concept of integrating Western and Chinese medicine has gained acceptance, improving the overall medical level. Therefore, research into contemporary Chinese medicine hospitals is crucial to enhance their technical efficiency and contribute to the development of the healthcare system. The state's support for TCM hospitals promotes public health, traditional culture, and national economic growth. Further research on TCM hospital efficiency can identify issues and offer timely recommendations, ensuring the continued growth and innovation of TCM hospitals.

### 4.4 Static analysis

#### 4.4.1 Data envelopment method efficiency analysis

**Figure 3:**
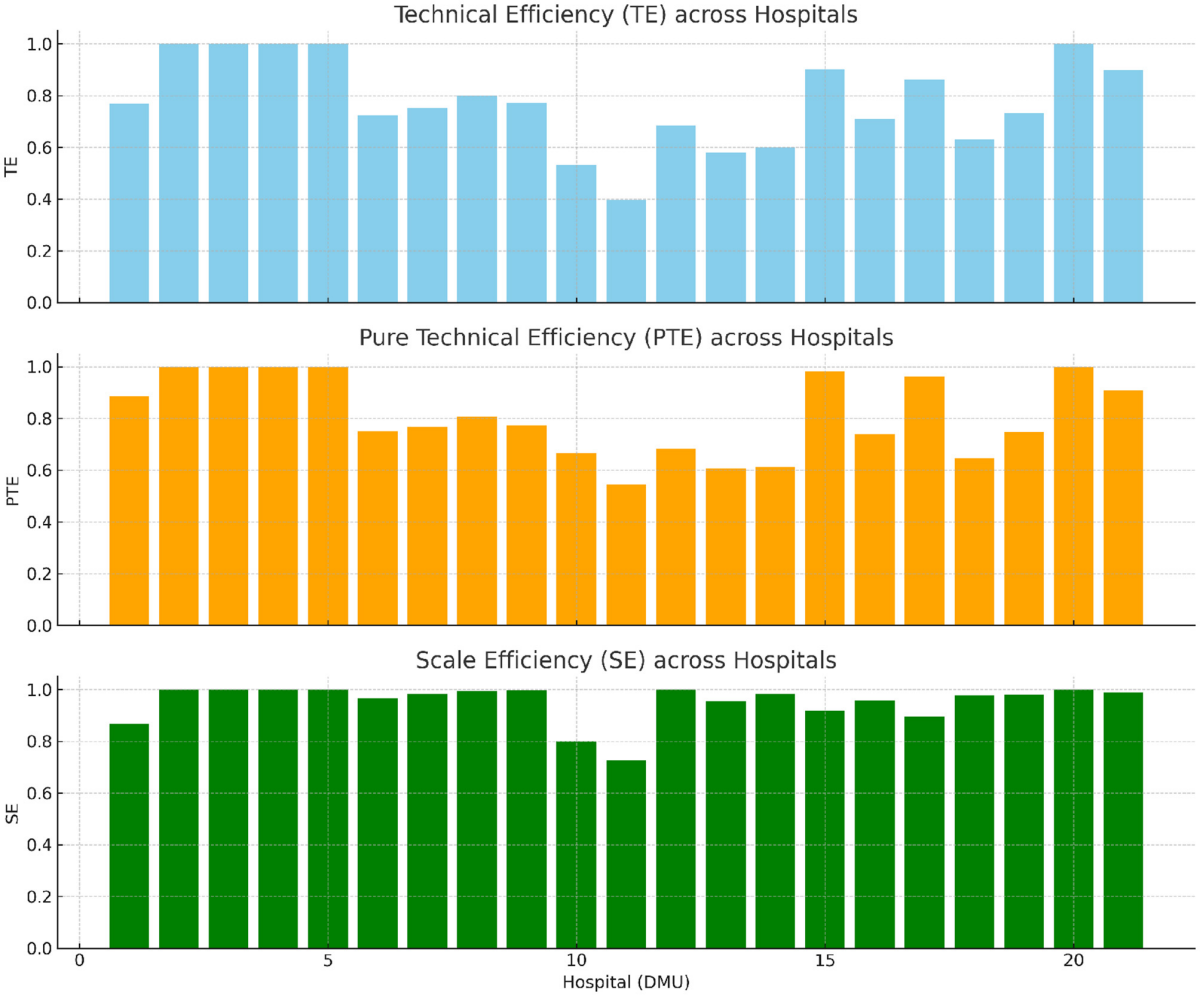
Bar plots of TE, PTE, and SE across 21 TCM hospitals in Hebei Province.

We bring the input and output indicators into the DEAP software, and after some processing, we can get the following results, which are shown in Table 6 and Figure 3. Through the DEA-CCR and BCC methods, we can derive the stages of development of TE, PTE, SE, and the corresponding DMU size gains of the 21 public TCM hospitals, which are further analyzed in the following section.

**Table 6:** DEA efficiency analysis of 21 TCM hospitals in Hebei Province.

**DMU**	**TE**	**PTE**	**SE**	**Gain or loss of scale**
1	0.76942	0.886734	0.8677	Irs
2	1	1	1	-
3	1	1	1	-
4	1	1	1	-
5	1	1	1	-
6	0.723235	0.748482	0.966269	Irs
7	0.752896	0.765806	0.983143	Irs
8	0.801318	0.806718	0.993307	Drs
9	0.771972	0.77405	0.997316	Irs
10	0.531185	0.664818	0.798994	Irs
11	0.395515	0.544911	0.725833	Irs
12	0.682265	0.682299	0.99995	Irs
13	0.578904	0.606148	0.955053	Irs
14	0.600521	0.611689	0.981742	Irs
15	0.901371	0.982562	0.917368	Irs
16	0.708786	0.740304	0.957426	Irs
17	0.861391	0.961406	0.89597	Irs
18	0.631934	0.646362	0.977679	Irs
19	0.733149	0.747304	0.981058	Irs
20	1	1	1	-
21	0.898655	0.90896	0.988662	Irs
Average	0.778215	0.813264	0.951784	-

Comprehensive technical efficiency (TE) is a key indicator for measuring the overall operation of Chinese medicine hospitals in Hebei Province. It reflects the effectiveness of resource allocation and is used to evaluate resource utilization and hospital performance (54). As shown in Table 6, the average TE of TCM hospitals is 0.778. Five hospitals, accounting for 23%, exhibit effective TE, indicating good resource utilization and efficient operation. These hospitals benefit from strong supervisory mechanisms and smooth communication between management levels. However, 16 hospitals, or 76%, have ineffective TE, highlighting the need for improvement in their operations. Most hospitals have compensable operational inefficiencies, with TE values ranging from 0.6 to 0.8, indicating low overall efficiency. Further analysis of pure technical efficiency (PTE) and scale efficiency (SE) is needed. The lowest TE values belong to DMU11 and DMU10, both county-level TCM hospitals, suggesting a correlation between operational efficiency and geographic factors. These factors will be removed through stochastic frontier analysis in future steps.

Pure technical efficiency (PTE) measures input productivity at the optimal scale, reflecting the effectiveness of resource management (55). Hospitals with low PTE are far from achieving optimal efficiency. According to Table 6, the average PTE of TCM hospitals is 0.813. 5 hospitals (23%) have effective PTE, indicating maturity in management and technology, while 16 hospitals (76%) have ineffective PTE, showing significant room for improvement. The majority of these hospitals have PTEs between 0.6 and 0.8, suggesting weaknesses in management and technology. Enhancing the training of TCM technicians and improving hospital management practices can lead to quicker improvements. By optimizing resource use, strengthening process management, and implementing scientific methods, the technical efficiency of TCM hospitals can be enhanced (56, 57).

Scale efficiency (SE) reflects the development stage of Hebei Provincial Hospital of Traditional Chinese Medicine. While continuous investment in fixed assets may negatively impact hospital operations, the theory of returns to scale suggests that efficiency rises initially and then declines. To optimize investment, it is necessary to reduce input for declining returns (DRS) and increase input for increasing returns (IRS) (58). Table 6 shows that 5 out of 21 hospitals are efficient and at the fixed SE stage (23.8%), one hospital has decreasing SE (4.8%), and 15 hospitals (71.4%) have increasing SE. The data indicate most hospitals are improving their SE, suggesting the need for further investment by the government to enhance operational efficiency. However, some hospitals are experiencing diminishing returns, signaling issues such as resource wastage, internal management problems, and miscommunication (59).

According to Appendix Table A4, we can see that the average values of TE, PTE, and SE of TCM hospitals are high, but the minimum values are unstable, and the smallest comprehensive efficiency is already below 0.4, which indicates that the development of TCM hospitals is very unbalanced. This is partly due to environmental and location factors, which need to be determined and eliminated by parametric methods.

According to the development of the efficiency of TCM hospitals, those with efficiency scores greater than 0.9 are defined as the high band, 0.6 to 0.9 as the medium efficiency band and below 0.6 as the low efficiency band. From the analysis of the efficiency bands in Table 7, it can be seen that the majority of TCM hospitals are in the high and middle bands of the efficiency scores. There are 6 TCM hospitals in the high band of TE, 12 in the middle band, and 3 in the low band; 8 TCM hospitals in the high band of PTE, 12 in the middle band, and 1 in the low band; and 17 TCM hospitals in the high band of SE, and 4 in the middle band. In general, there are relatively few TCM hospitals in the low-efficiency band. The efficiency levels provide a practical reference for hospital administrators and policymakers to focus on performance improvement efforts. Hospitals in the low-efficiency band may require capacity building programs or external support, while high-efficiency hospitals could serve as benchmarks for best practices. Moreover, identifying hospitals in the middle band offers opportunities for transitional support strategies to elevate them to higher efficiency levels.

**Table 7:** Efficiency value of Hebei Provincial TCM hospital by score bands.

**Segment**	**Combined efficiency**		**Pure technical efficiency**		**Scale efficiency**	
	**N**	**Average**	**N**	**Average**	**N**	**Average**
High band (0.9 ~)	6	0.984	8	0.982	17	0.982
Middle band (0.6 ~ 0.9)	12	0.745	12	0.723	4	0.823
Low band (0 ~ 0.6)	3	0.502	1	0.555		

According to the National Health Commission's classification, Chinese medicine hospitals in Hebei Province are categorized based on bed numbers: small (below 900 beds), medium (900–1,200 beds), and large (above 1,200 beds). Appendix Table A5 shows that 3 medium and small-sized hospitals (14.3%) have average efficiencies of 0.802, 0.749, and 0.845; 6 medium-sized hospitals (28.6%) have average efficiencies of 0.855, 0.891, and 0.939; and 12 large-sized hospitals (57.14%) have average efficiencies of 0.837, 0.897, and 0.856. The TE for hospitals of these sizes averages around 0.85.

The data reveal that medium-sized hospitals (900–1,200 beds) have the highest efficiencies, followed by large-sized hospitals (over 1,200 beds) and small-sized hospitals (under 900 beds). This suggests that maintaining around 1,000 beds optimizes hospital efficiency, aligning with National Health Commission guidelines. Public hospitals should avoid the uncontrolled expansion of fixed assets like beds, as it can waste resources and decrease efficiency.

#### 4.4.2 SFA efficiency analysis

SFA, or stochastic frontier analysis, introduces influencing factors through parametric methods and is able to link the correlation between multiple inputs and outputs, which is applicable to the efficiency evaluation of various fields. Through the SFA model, we can get the specific impact of the external environment and statistical noise on the efficiency of hospitals, and in the subsequent study, the random factors are eliminated, and only the relevant factors of the hospital's own management and inputs are retained, to provide a more objective and realistic evaluation of the TE of each type of hospital (60). According to the previous section on indicator selection, we choose the logarithmic values of these two variables for comparative regression analysis because the government's financial input and science and education-related inputs are externally determined, and the data are accurate and easy to obtain, and have nothing to do with the hospital's own management and configuration factors, and for a number of other reasons.

The regression results are shown in Table 8a. Based on the λ-values, we can see that the equations for slack variables 1, 2, and 3 are significant, which suggests that the number of healthcare workers, fixed assets, and number of beds in operation in Chinese medicine hospitals are affected by both external environmental factors and management factors. The λ values of the equations for slack variables 1 and 3 are at a certain distance from 1, indicating that random noise also affects the input variables and that management and random factors need to be separated. In the SFA equation for slack variable 3, the logarithm of financial and technological inputs is “+” and is significant at the 1% level, which means that increasing financial and technological inputs has a positive effect on the investment in fixed assets of TCM hospitals and that an increase in the number of resources needed to produce the same output does not help to increase TE. In the regressions of input variables 1 and 2, the logarithm of scientific research inputs is (–), and the coefficient in the regression for healthcare technicians is significant at the 1% level. This indicates that investment in science and education can increase the utilization of human resources; in the regression of input variable 2, the logarithm of financial input is (–), and the coefficient of the regression of the number of beds at the end of the year is significant at 1%, which indicates that the higher financial input can increase the utilization of beds, further proving the effect of the environmental variables on the efficiency of the hospitals.

**Table 8:** SFA regression results and influence of environmental factors on the input variables.

**Variables**	**Number of health technicians (slack variable 1)**	**Average number of beds (slack variable 2)**	**Fixed assets (slack variable 3)**
**(a) SFA regression analysis of operational efficiency of TCM hospitals in Hebei Province**			
β	1.24E+01	-6.37E+01	-3.52E+03
	(21,124.02)	(79.538)	(4.71E+04)
*ln*(*financial input*)	4.37E+03^***^	-2.86E+04^***^	5.43E+05^***^
	(1.89E+01)	(6.49E+03)	(1.38E+01)
*ln*(*scientific and technical inputs*)	–1.53E+03^***^	–1.46E+03	3.01E+04^***^
	(2.03E+01)	(1.22E+05)	(3.33E+02)
σ2	1.74E+05^***^	1.55E+04^***^	3.48E+08^***^
	(1.000)	(1.000)	(1.000)
γ	0.930^***^	0.998^***^	0.938^***^
	(0.003)	(0.000)	(0.002)
log-likelihood	-1.03E+02	-3.08E+02	-2.51E+02
LR	8.42E+00^***^	9.62E+00^***^	9.38E+00***
**(b) Influence of environmental factors on the input variables of TCM hospitals in Hebei Province**.			
Fiscal inputs	-	+	-
Science education inputs	+	/	-

t-statistics in parentheses.

^***^ *p* < 0.01, ^**^p < 0.05, ^*^p < 0.1.

Table 8b indicates that a “+” sign means the input indicator positively impacts resource utilization, thereby improving technical efficiency. Further analysis shows that in Hebei Province's Chinese medicine hospitals, fixed asset input and bed numbers are significantly influenced by regional factors, affecting the accuracy of internal efficiency measurements. The input of health and technical personnel is influenced by random factors. Local financial conditions impact the allocation of fixed assets and bed numbers, thus affecting hospital efficiency. This aligns with our analysis, as central cities, with more financial and educational resources, experience higher efficiency in their local hospitals.

Except for bed input, financial inputs negatively affect the other efficiency intermediaries, suggesting that continued subsidies may expand inputs without improving hospital efficiency. Furthermore, medical and nursing personnel are still scarce in TCM hospitals. Investing in scientific education can enhance the technical efficiency of TCM hospitals. Cultivating more TCM practitioners and improving bed utilization will help meet the growing demand for TCM services.

#### 4.4.3 Super-SBM efficiency analysis

One limitation of the DEA model is that it cannot rank multiple effective DMUs (efficiency = 1). To address this, we used SFA regression analysis to eliminate external environmental factors and noise, then conducted Super-SBM analysis on the SFA-adjusted input data. The technical efficiency results for TCM hospitals are shown in Table 9. From 2017, DMU4, DMU2, DMU8, DMU3, and DMU7 hospitals were deemed efficient, with DMU4 being the most efficient, followed by DMU2 and DMU8. In 2018, DMU2 hospitals were rated the most efficient, followed by DMU4 and DMU5. This approach allows for year-to-year analysis of efficiency changes, facilitating recommendations and evaluations for each hospital.

**Table 9:** Super-SBM efficiency analysis for Hebei Provincial TCM hospital.

**DMU**	**2017**		**2018**	
	**Efficiency**	**Rank**	**Efficiency**	**Rank**
DMU1	0.769	11	0.846	8
DMU2	1.072	2	1.099	1
DMU3	1.026	4	1.020	4
DMU4	1.086	1	1.081	2
DMU5	0.964	7	1.057	3
DMU6	0.669	12	0.642	13
DMU7	1.023	5	0.674	12
DMU8	1.032	3	0.709	10
DMU9	0.589	14	0.669	11
DMU10	0.521	16	0.530	18
DMU11	0.378	19	0.405	21
DMU12	0.620	13	0.548	17
DMU13	0.332	21	0.461	19
DMU14	0.339	20	0.452	20
DMU15	0.897	9	0.935	6
DMU16	0.537	15	0.606	14
DMU17	0.961	8	0.906	7
DMU18	0.449	18	0.587	16
DMU19	0.513	17	0.600	15
DMU20	0.969	6	1.000	5
DMU21	0.799	10	0.812	9

Compared to the traditional DEA method (see TE values in Table 6), DMU10 and DMU11 county hospitals ranked last in technical efficiency. After adjusting for external and random factors, DMU10 moved up three places, indicating that local financial and educational conditions contributed to better efficiency. Although there is still room for improvement, DMU10 is no longer last. Conversely, DMU11 remained at the bottom in 2018, highlighting that its poor technical efficiency is due to internal management issues rather than external factors.

**Figure 4:**
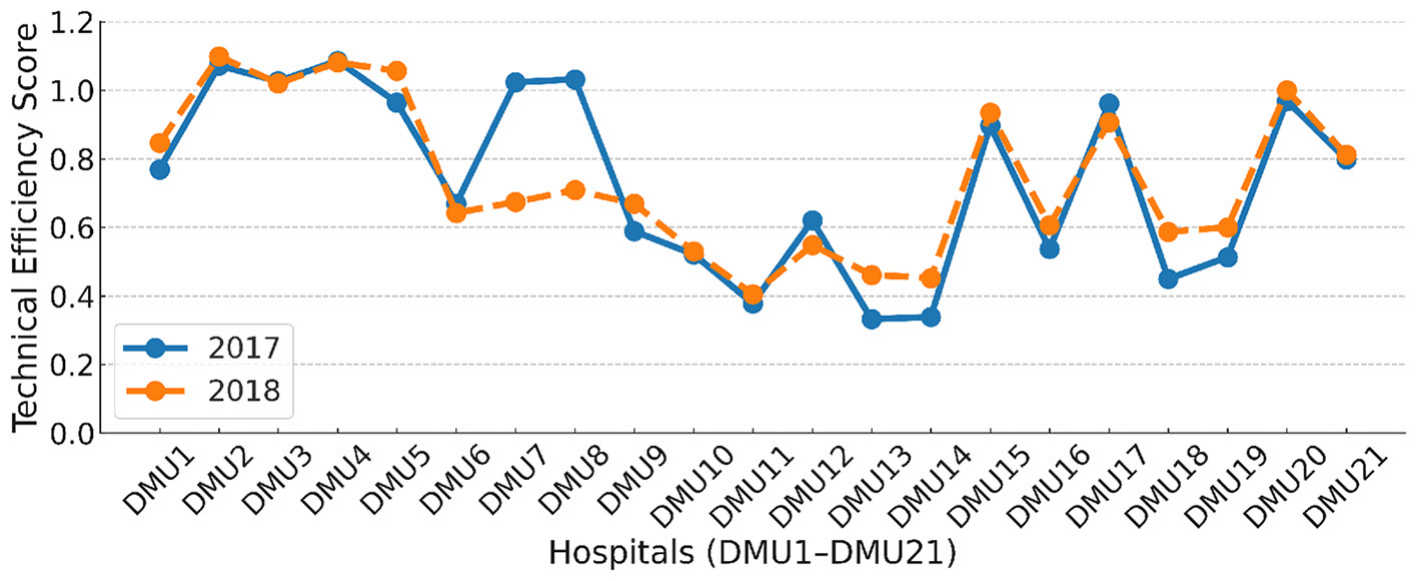
Technical efficiency scores of 21 TCM hospitals in Hebei Province for 2017 and 2018.

Through Figure 4, we analyze the efficiency of 21 TCM hospitals in Hebei Province in a 2-year comparison. We can see that the technical efficiency of 14 TCM hospitals has been slightly improved, the efficiency of 2 TCM hospitals has a huge drop, and the other hospitals remain in their reasonable range of stable fluctuations, which indicates that the technical efficiency of TCM hospitals as a whole did not get big progress in 2018 and that the government's relevant input policies still need to be improved in 2018, especially because some hospitals have a huge drop in TE, which is a great waste of resources and management deficiencies that must be brought to the attention of regulators. In particular, In particular, DMU6 and DMU7 experienced significant declines in technical efficiency, dropping from rank 12 to 13 and from rank 5 to 12, respectively. These shifts may reflect local mismanagement, resource misallocation, or disruptions associated with the ongoing implementation of the hierarchical medical reform, such as patient diversion or cost-sharing mechanisms. Conversely, DMU10 rose three positions in ranking after adjustment, suggesting that improved local conditions—such as financial input, administrative oversight, or staff retention—may have begun to alleviate prior inefficiencies. Such rank changes, when viewed alongside environmental adjustments, provide indirect evidence on the differential institutional responses to provincial policies and internal constraints. This also highlights the need for localized management strategies beyond uniform policy incentives.

### 4.5 Dynamic analysis

#### 4.5.1 Malmquist efficiency analysis

The modified three-stage method accurately measures the technical efficiency of hospital units, eliminating the impact of environmental and random factors. This allows us to assess efficiency based solely on internal decision-making, offering a more reliable measure. However, this study is static and does not capture dynamic changes in efficiency for Chinese medicine hospitals in Hebei. To address this, we applied the Malmquist model, re-analyzing the Malmquist index with input data from the corrected three-stage model. This allows us to evaluate the dynamic technical efficiency of Hebei's public Chinese medicine hospitals from 2014 to 2018. The Malmquist model measures the growth rate of total factor productivity (TFP). When TFP > 1, efficiency improves, and when TFP < 1, efficiency declines:


(17) $$\begin{array}{l} MPI=TCI\times TECI=TCI\times PTECI\times SECI, \end{array}$$

where Malmquist Productivity Index (MPI) represents the total factor productivity change index, TECI is the technical efficiency change index, TCI is the technical progress change index, PTECI is the pure technical efficiency change index, and SECI is the scale efficiency change index. This formula reveals not only year-to-year changes in efficiency but also the technological development of hospitals (61).

We used the MPI model to measure and decompose the total factor productivity (TFP) change in TCM hospitals across regions over time, as shown in Table 10. The MPI index for Hebei's TCM hospitals decreased slightly over five years, with an average value of 0.983, representing a 1.7% decline. This change results from a 5.8% decrease in technological change and a 1.5% increase in technical efficiency. The increase in technical efficiency is driven by a 0.2% rise in pure technical efficiency (PTECI) and a 0.7% rise in scale efficiency (SECI).

**Table 10:** Malmquist index and decomposition of public TCM hospitals in Hebei Province, 2014–2018.

**DMU**	**14–15**	**15–16**	**16–17**	**17–18**	**Average**
	**(Phase 1)**	**(Phase 2)**	**(Phase 3)**	**(Phase 4)**	
MPI	0.962	1.031	0.983	0.957	0.983
TCI	1.116	0.887	0.962	0.957	0.942
TECI	0.868	1.170	1.022	1.000	1.015
PTECI	0.926	1.090	1.012	0.989	1.002
SECI	0.938	1.069	1.010	1.012	1.007

The decline in productivity is mainly due to technological regression, with minimal improvement from technological efficiency. Despite internal efforts to optimize operations, technological regression driven by external factors is the primary cause. Specifically, technical change increased by 11.6% in the first period but decreased by 11.3% in the second, and by 3.8% and 4.3% in the third and fourth periods, respectively. This is likely related to the decline in TCM hospitals' technical levels, the migration of TCM professionals, and imbalanced incentives. The sustained drop in TCI coincides with the implementation of the *Development Plan for TCM Health Services (2015–2020)*, which prioritized infrastructure construction, integration of TCM into elderly care, and prevention-focused services. These priorities temporarily redirected investment and institutional focus toward primary-level development rather than hospital-based technological advancement. The rate of technical efficiency change increased by 17.0% in the second period after a 13.2% drop in the first, and then increased by 2.2% and remained unchanged in the third and fourth periods. This fluctuation in TECI aligns with the phased rollout of Hebei's *Hierarchical Medical System Implementation Guidelines (2015)*, which began in 2015 and achieved province-wide coverage by 2017. By reshaping referral patterns and redistributing patient flow, the reform indirectly affected the case mix and staffing needs of public TCM hospitals, contributing to shifts in technical efficiency alongside other operational adjustments such as depreciation accounting and ICDS expansion. The spike in technical efficiency in the second period may be due to changes in depreciation expense accounting in 2016 and the expansion of the Integrated Care Delivery System (ICDS) in 2015, which required more nursing staff. Lower bed utilization and higher labor costs further reduced operational efficiency (62). From 2016–2018, despite integrated care reforms, technical efficiency showed minimal real improvement beyond accounting effects.

According to Appendix Table A6, the technical efficiency change index (TECL) shows that from 2014 to 2018, DMU4, DMU6, DMU8, and DMU9 experienced a decline in technical efficiency. For DMU4 and DMU6, the decrease was mainly due to a drop in pure technical efficiency, with resource waste from mismanagement being the key factor. For DMU8 and DMU9, the decline in efficiency resulted from both reduced pure technical efficiency and scale efficiency. By decomposing efficiency changes, we can identify the factors affecting hospital technical efficiency and service production over time, allowing for targeted interventions to improve the overall operational efficiency of TCM hospitals (63).

#### 4.5.2 Tobit regression

In the previous section, we used the modified three-stage model to measure the technical efficiency of Hebei Provincial Hospital of Traditional Chinese Medicine (HBHM) and analyzed the results. However, as this is a static analysis, we applied the Malmquist index model to examine dynamic efficiency, breaking down total factor productivity change into technology change, technical efficiency change, pure technical efficiency change, and scale efficiency change. Still, we cannot quantitatively determine which specific input and output factors affect the technical efficiency of Hebei TCM hospitals. Therefore, in this section, we apply the Tobit model to regressively analyze these factors (64).

The Tobit model, a restricted dependent variable model, is suitable as the technical efficiency values derived from the modified three-stage model are continuous and greater than 0. Traditional OLS regression would yield biased results, so we use maximum likelihood estimation with the Tobit model.

We selected variables based on operational and financial factors affecting hospital efficiency. After removing environmental influences in the three-stage model, we considered data availability, representativeness, and comprehensiveness, including operational indicators like hospital scale, medical services, and financial factors such as income structure, development capacity, asset operation, and cost management (65).

Operational factors include the number of health and technical personnel, beds, and depreciation expense, reflecting hospital scale. Bed utilization, outpatient numbers, and average length of stay represent service quality, with higher outpatient numbers indicating greater patient acceptance and a shorter length of stay reflecting better medical technology.

**Figure 5:**
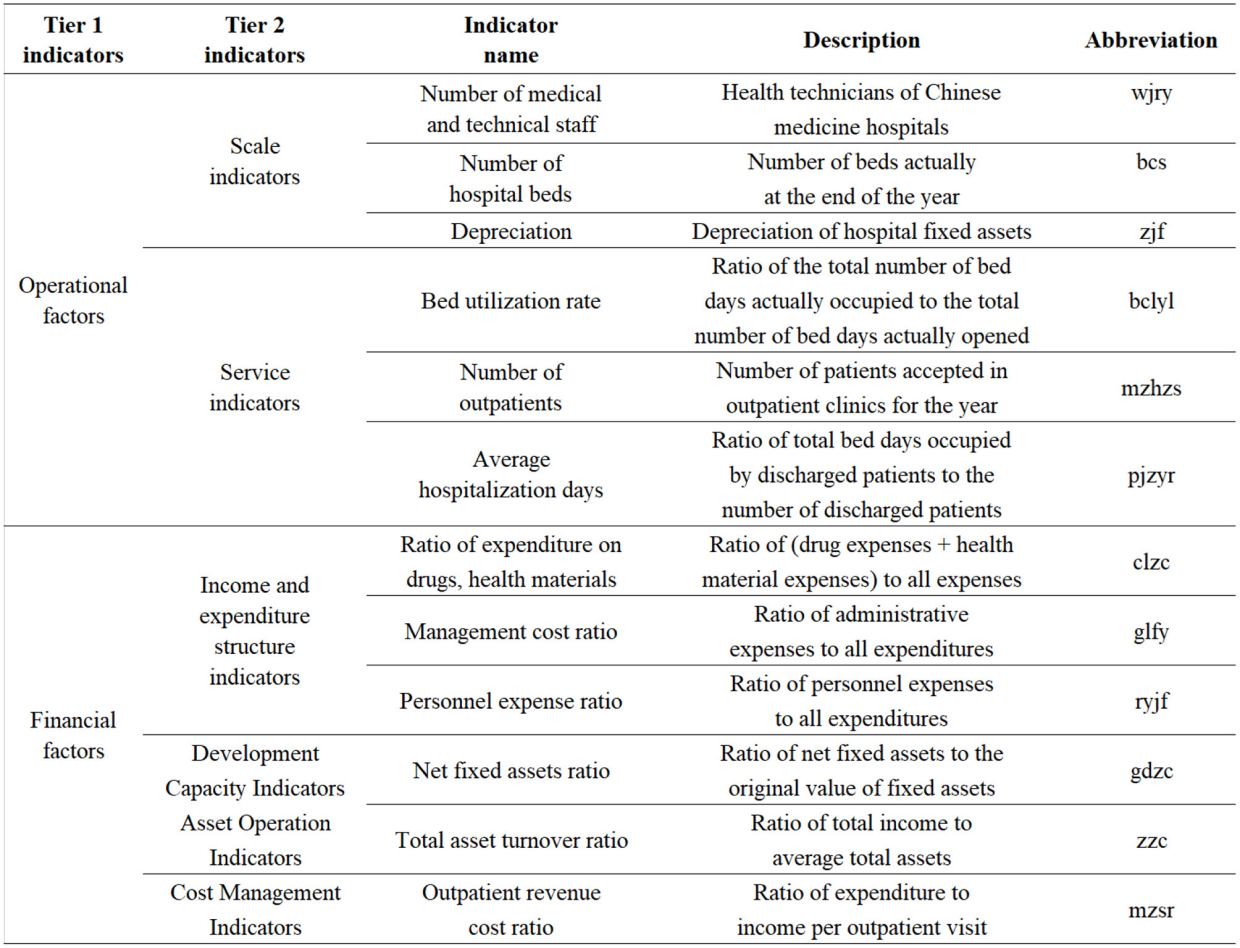
Tobit regression indicator system for analyzing the factors influencing technical efficiency of traditional TCM hospitals in Hebei Province.

Financial factors include drug and sanitary material expenditure ratios, management expense ratios, personnel expense ratios, net fixed asset ratios, total asset turnover ratios, and outpatient income cost ratios. Higher ratios suggest poor internal management, while lower personnel expense ratios reflect better resource allocation. Net fixed asset ratios show asset condition, while total asset turnover ratios indicate operational strength. The outpatient income cost ratio reflects cost management, with higher ratios signaling better resource utilization and internal control. See Figure 5 for specific indicators.

In this paper, the value of the technical efficiency of TCM hospitals in Hebei Province obtained by the third stage SBM model is used as the explanatory variable, and the above indicators are used as the dependent variables. To avoid coefficient bias, the Tobit regression model is used to analyze the factors influencing the technical efficiency of TCM hospitals in Hebei Province in 2014–2018, and the specific regression equations are as follows:


(18) $$\begin{array}{l} y_{\text{it}}=\beta_{0}+\beta_{1}wjry_{it}+\beta_{2}\text{bc}{\text{s}}_{\text{it}}+\beta_{3}\text{zj}{\text{f}}_{\text{it}}+\beta_{4}\text{bcly}{\text{l}}_{\text{it}}+\beta_{5}\text{mzhz}{\text{s}}_{\text{it}}+\\ {\;\beta }_{6}\text{pjzy}{\text{r}}_{\text{it}}+\beta_{7}\text{clz}{\text{c}}_{\text{it}}+\beta_{8}\text{glf}{\text{y}}_{\text{it}}+\beta_{9}\text{ryj}{\text{f}}_{\text{it}}+\beta_{10}\text{gdz}{\text{c}}_{\text{it}}\\ \;+\beta_{11}\text{zz}{\text{c}}_{\text{it}}+\beta_{12}\text{mzs}{\text{r}}_{\text{it}}+\varepsilon_{\text{it}}. \end{array}$$

The relevant data from 2014–2018 was brought into the software for Tobit regression, and based on the modeling assumptions above, we obtained the regression results as shown in Appendix Table A7.

The panel data regression results indicate that five variables significantly affect the technical efficiency of Hebei Provincial Hospital of Traditional Chinese Medicine at the 5% level: depreciation expense, bed utilization rate, management expense ratio, personnel expense ratio, and total asset turnover ratio. Depreciation expense and management expense ratio negatively affect technical efficiency, while bed utilization rate, personnel expense ratio, and total asset turnover ratio positively impact it. Additionally, the number of beds negatively influences technical efficiency at the 10% significance level.

Regarding operational factors, only depreciation expense and bed utilization rate were statistically significant at the 5% level. Depreciation expense, which reflects the size of fixed assets, is negatively correlated with technical efficiency. Larger fixed assets lead to higher depreciation costs, which in turn reduce technical efficiency. This suggests that Hebei hospitals have older fixed assets, and future investment in new equipment could enhance service and efficiency (66). Bed utilization rate has a positive effect, as improving this rate enhances hospital service efficiency, reduces average hospitalization time, and improves technical efficiency. The negative coefficient for the number of beds at the 10% significance level suggests that further expansion of bed numbers could harm overall efficiency. This may be due to Hebei's proximity to Beijing and Tianjin, where people tend to seek medical care, causing a bed oversupply in Hebei.

For financial factors, the management expense ratio, personnel expense ratio, and total asset turnover ratio were significant at the 5% level. The management expense ratio negatively impacts technical efficiency, indicating poor internal control and excess management personnel. The personnel expense ratio has a positive correlation with technical efficiency, suggesting that increasing salaries for health technicians can enhance efficiency. The total asset turnover ratio, however, negatively affects technical efficiency, implying that improving asset utilization and focusing on income generation could boost efficiency. This negative association may appear counterintuitive, but it reflects the fact that some hospitals with higher turnover ratios tend to prioritize revenue generation over optimizing internal processes or investing in long-term improvements. In such cases, high asset turnover could be driven by excessive reliance on outpatient volume or service expansion, which may not correspond to improved technical efficiency under the DEA framework. Moreover, these hospitals may be under pressure to meet fiscal performance targets, potentially at the cost of sustainable operational efficiency. Therefore, hospitals should optimize asset use, maintain current investment levels, improve services, and promote Chinese medicine to attract local patients.

## 5 Conclusion

Our findings match with recent research on hospital efficiency across Chinese provinces. For example, Sun et al. (67) and Huang et al. (68) found technical inefficiencies and technological stagnation as key drivers of productivity loss in public hospitals in Fujian and Guangxi, similar with our results from Hebei's TCM hospitals. Compared to earlier studies using single-stage DEA (69), our use of a three-stage DEA and Malmquist productivity index provides more accurate decomposition of efficiency components by adjusting for environmental factors and capturing dynamic trends. Furthermore, by focusing on TCM hospitals, a less-explored segment, our analysis contributes a novel institutional perspective to frontier efficiency studies. These methodological and empirical improvement help refine policy targeting and resource optimization in healthcare systems.

This study integrates DEA with SFA to account for environmental influences, introduces the super-SBM model to refine efficiency measurement, and evaluates 21 hospitals in Hebei Province. A static analysis was conducted, followed by an MPI-based dynamic analysis from 2014 to 2018, and a Tobit model was used to identify efficiency factors.

The static results revealed moderate average levels of technical and pure technical efficiency, with relatively high scale efficiency. Most inefficiencies stemmed from internal operational issues rather than hospital size. Among hospitals, 4.8% showed decreasing returns to scale, 71.4% increasing returns, and 23.8% were scale-efficient. These findings highlight the need for targeted resource allocation to enhance efficiency and prevent waste due to mismanagement, corruption, and overprescription (70).

Without accounting for environmental factors, efficiency peaks when hospitals maintain around 1,000 beds, suggesting that unregulated expansion may lead to inefficiencies. The second-stage SFA analysis confirms that environmental and locational factors significantly impact efficiency, with financial investment alone proving insufficient, while enhancing practitioner training can improve performance.

The third-stage super-SBM analysis reveals little efficiency improvement over 2 years, with some hospitals experiencing declines due to resource misallocation and poor management, warranting regulatory attention. Changes in efficiency rankings reflected the varying influence of external funding and internal administration across hospitals.

MPI results indicate a slight decline in efficiency from 2014–2018, primarily driven by a slowdown in technological progress, despite marginal improvements in technical efficiency. This indicates that long-term efficiency gains in TCM hospitals may depend more on innovation and technology adoption than on scale adjustment alone. These findings suggest technological regression in TCM hospitals, with limited efficiency-driven growth, necessitating further analysis of individual hospitals.

Tobit regression analysis identifies key efficiency factors: depreciation expenses, management expense ratios, and increased bed capacity negatively impact efficiency, while bed utilization rates, personnel expense ratios, and total asset turnover ratios contribute positively. These findings underscore the importance of strategic resource allocation, financial planning, and management optimization to enhance hospital efficiency in Hebei Province.

To address inefficiencies, especially those driven by technological stagnation and mismanagement, several targeted strategies are recommended. First, provincial health authorities should introduce performance-based funding mechanisms to reward hospitals that demonstrate continuous efficiency improvements. Second, targeted financial subsidies and technical assistance should be prioritized for county-level hospitals exhibiting persistent inefficiencies. Third, dedicated training programs for TCM practitioners and administrators should be developed. Lastly, policy support should encourage rationalization of the hospital scale by guiding bed allocation and curbing inefficient expansions. At the hospital level, institutionalizing internal performance monitoring systems and enabling data-driven managerial decision-making could further sustain efficiency gains. These recommendations are grounded in the efficiency theory underpinning DEA and SFA models, which emphasize the role of managerial capacity and resource alignment in improving performance outcomes in healthcare organizations.In summary, this study provides robust empirical evidence on the technical efficiency and factors influencing TCM hospitals in Hebei province. By integrating DEA, SFA, and MPI methodologies, it offers a replicable framework for efficiency evaluation and contributes to evidence-based policy formulation aimed at optimizing hospital performance in similar healthcare contexts.

## Data Availability

The data analyzed in this study is subject to the following licenses/restrictions: The raw data supporting the conclusions of this article will be made available by the authors on request. Requests to access these datasets should be directed to Ziyin Liao, ziyin722@whu.edu.cn.
