# Supplementary material for: Evaluating technical efficiency and influencing factors in traditional Chinese medicine hospitals: evidence from Hebei Province, China
Source: Front Public Health. 2025 Sep 10;13:1621755. doi: 10.3389/fpubh.2025.1621755 (PMC12457436; doi:10.3389/fpubh.2025.1621755)
Supplement: Supplementary file 1 [file Table_1.docx]

**Appendix**

**Table A1****.** Input and output variables

|  | Variables | Measurement indicators |
| --- | --- | --- |
| Input Variables | Number of health care  technical employees  Number of beds  Fixed Assets | Practitioners of Chinese medicine and  other healthcare workers  Actual number of beds in operation  at the end of the year  Buildings and equipment |
| Output Variables | Number of outpatients  Number of inpatients  Revenue from visits  Income from hospitalization | Number of outpatients in the year  Number of inpatients in the year  Revenue from Consultation  Inpatient Revenue |

**Table A2.** Staffing of general hospitals and hospitals of traditional Chinese medicine in Hebei

| Indicators |  | General hospitals | Chinese hospitals |
| --- | --- | --- | --- |
| Active employees  (persons) | Average  Standard Deviation  Maximum value  Minimum value | 2,166.57  1,036.46  4,536.00  689 | 1,282.81  493.19  2,153.00  587 |
| Healthcare technology employees (persons) | Average  Standard Deviation  Maximum value  Minimum value | 1,782.86  894.86  3,451.00  548 | 1,086.14  407.78  1,842.00  411 |
| Management workers  (persons) | Average  Standard Deviation  Maximum value  Minimum value | 201.71  115.97 553 57 | 91.86  46.25  212  20 |
| General work  employees (persons) | Average  Standard Deviation  Maximum value  Minimum value | 182  92.96  532  64 | 104.81  83.25 411 24 |

**Table** **A3.** Staff ratio of general hospitals and Chinese medicine hospitals in Hebei Province

| Indicators | General hospitals | Chinese hospitals |
| --- | --- | --- |
| The proportion of health care technical staff to active staff (%) | 82.29 | 84.67 |
| Proportion of managerial staff in the workforce (%) | 9.31 | 7.16 |
| General staff as a  percentage of staff (%) | 8.4 | 8.17 |

**Table A4.** Descriptive analysis of efficiency value of traditional Chinese medicine hospitals in Hebei Province

| Efficiency | N | Minimum | Maximum | Average |
| --- | --- | --- | --- | --- |
| Comprehensive efficiency | 21 | 0.396 | 1 | 0.778 |
| Pure technical efficiency | 21 | 0.545 | 1 | 0.813 |
| Scale efficiency | 21 | 0.726 | 1 | 0.951 |

**Table A5.** Efficiency values of Chinese medicine hospitals in Hebei Province by hospital size

| Number | Sample | Integrated operational | Pure technical | Efficiency |
| --- | --- | --- | --- | --- |
| of beds | size | efficiency | efficiency | of scale |
| N < 900 | 3 | 0.802 | 0.749 | 0.845 |
| 900 ≤ N < 1200 | 6 | 0.855 | 0.891 | 0.939 |
| N ≥ 1200 | 12 | 0.837 | 0.897 | 0.856 |

**Table A6.** Statistical table of MPI decomposition of public traditional Chinese medicine hospitals in Hebei Province, 2014-2018

| DMU | MPI | TCL | TECI | PTECI | SECI |
| --- | --- | --- | --- | --- | --- |
| DMU1 | 0.992 | 1.002 | 1.016 | 0.978 | 1.038 |
| DMU2 | 0.986 | 1.002 | 1.009 | 0.969 | 1.033 |
| DMU3 | 1.027 | 0.997 | 1.045 | 1.016 | 1.035 |
| DMU4 | 0.955 | 1.002 | 0.971 | 0.964 | 1.003 |
| DMU5 | 0.998 | 0.997 | 1.001 | 1.000 | 1.000 |
| DMU6 | 0.982 | 0.993 | 0.996 | 0.989 | 1.004 |
| DMU7 | 1.021 | 1.003 | 1.012 | 1.038 | 0.972 |
| DMU8 | 0.936 | 0.975 | 0.965 | 0.967 | 0.999 |
| DMU9 | 0.965 | 0.986 | 0.996 | 0.999 | 0.997 |
| DMU10 | 0.982 | 0.989 | 1.010 | 1.005 | 1.000 |
| DMU11 | 0.992 | 0.985 | 1.026 | 1.034 | 0.993 |
| DMU12 | 0.940 | 0.950 | 1.000 | 1.000 | 1.000 |
| DMU13 | 0.990 | 0.953 | 1.044 | 1.042 | 1.002 |
| DMU14 | 0.937 | 0.930 | 1.008 | 1.007 | 1.001 |
| DMU15 | 0.962 | 0.960 | 1.001 | 1.002 | 1.000 |
| DMU16 | 1.001 | 0.979 | 1.041 | 1.000 | 1.041 |
| DMU17 | 0.999 | 0.995 | 1.018 | 1.009 | 1.008 |
| DMU18 | 1.002 | 0.964 | 1.050 | 1.040 | 1.006 |
| DMU19 | 0.995 | 0.968 | 1.036 | 1.036 | 1.000 |
| DMU20 | 0.964 | 0.984 | 1.007 | 0.980 | 1.022 |
| DMU21 | 1.023 | 0.980 | 1.064 | 1.018 | 1.000 |

**Table A7.** Tobit model regression results

| Explanatory Variables | Coefficient | Stand error | t-value | P>\|t\| |
| --- | --- | --- | --- | --- |
| Number of health care workers | 2.83E-04 | 1.33E-03 | 0.21 | 0.831 |
| Number of hospital beds | -5.50E-03* | 2.84E-03 | -1.94 | 0.055 |
| Depreciation | -1.34E-10** | 6.15E-11 | -2.19 | 0.03 |
| Bed utilization rate | 7.48E-03*** | 2.02E-03 | 3.71 | 0 |
| Number of outpatients | -1.36E-07 | 5.71E-06 | -0.02 | 0.981 |
| Average hospitalization days | -2.88E-04 | 1.92E-03 | -0.15 | 0.881 |
| Expenditure ratio of drugs and sanitary materials | 5.10E-01 | 4.43E-01 | 1.15 | 0.252 |
| Management cost ratio | -4.07E-01*** | 1.49E-01 | -2.73 | 0.006 |
| Personnel expense ratio | 1.06E+00** | 1.02E+00 | 1.03 | 0.032 |
| Net fixed assets ratio | -2.07E-01 | 2.60E-01 | -0.8 | 0.428 |
| Total Asset Turnover Ratio | 1.77E-02** | 8.72E-03 | 2.03 | 0.045 |
| Outpatient revenue cost ratio | 2.30E-06 | 5.10E-06 | 0.45 | 0.657 |
